# The Third International Medical Congress

**Published:** 1873-11-01

**Authors:** 


MEDICAL EXAMINER.

*A Semi-Monthly Journal of Medical Sciences.*

EDITED BY

N. S. DAVIS, M. D., AND F. H. DAVIS, M. D

Chicago, Nov. 1st, 1873.

### EDITORIAL.

### THE THIRD INTERNATIONAL MEDICAL CONGRESS.

The late meeting of this body was held in Vienna, commencing September 1st, 1873, Prof. Carl Rakitansky presiding.

The following questions were discussed during the sessions:

1. The Question of Vaccination.
2. The Proposition of an International Law for the Prevention of Syphilis, and the Regulation of Prostitution.
3. The Question of Quarantine Measures against Cholera.
4. The Drainage of Cities.
5. The Proposition for the Adoption of ar International Pharmacopœia.
6. The Proposition for making Medical Studies Uniform, and, as a result, the Right of Practicing the Art freely in any Country.

Each question was assigned to a President and a Committee, who had the position of experts, to outline the subject for discussion, and to formulate the sentiments of the Congress for the ballot. These were printed and distributed before each session, as well as a daily bulletin containing the proceedings of the previous day.

Regarding the first question of Vaccination, the opinion was almost unanimous in favor of compulsory vaccination. Only three debaters, and that on insignificant grounds, declared against it. The opinion of the Congress, as declared by formal vote at the close of the discussion, was embodied in the following resolution:

"The Third International Medical Congress declares Vaccination indispensable, and desires its universal adoption."

As regards the second question, the Prevention of the Spread of Syphilis, considerable discussion took place, but no very definite opinion was reached. The meeting generally seemed to favor registration and police surveillance, according to the system in practice in most continental cities.

The question of Cholera Quarantine was decided as follows:

1. Land and sea quarantine to be abolished.

2. Sea quarantine to be temporarily continued.

3. An International Commission, for the study of the agencies which spread Cholera, and for eliminating them from commerce, as well as for finding rules which shall give greater protection than the present ones, is to be appointed.

Regarding the sanitary management of a city, as respects drainage and water supply, the referees reported as follows:

1. The purification and improvement of the city must be pointed out as an unavoidable demand from a sanitary point of view, and the study of the question is of the utmost importance.

2. For the conveyance of water to and from the houses, as well as channels for the surplus water, canalization is necessary in every city, and must be established in accord with hygienic demands.

3. The refuse of the inhabitants is to be conveyed away through rational means, in accordance with hygienic laws and the interests of the people.

4. In every case the circumstances of the city, the water-supply, the cost of engineering and management, the capability of paying, etc., must be all regarded.

5. As a rule, a good system in accord with hygienic demands can be made in a cheap and workable way.

6. The carrying off, at least partially, of fecal material, and of the greater part of the urine, is necessary.

7. Cities ought to engage in the discussion of these questions concerning the purification of the State and city, with the assistance of experts.

The following resolutions regarding an International Pharmacopœia were adopted:

1. The Congress recognizes the need of an International pharmacopœia. This must contain the most essential and best-known remedies, the most necessary excipients and corrigents, with a precise description of their qualities and method of preparation; the Latin language to be used in the text, and in the relations of the components the decimal system.

2. The Congress wishes that for the future the metrical system be used in prescribing.

3. The Congress intrusts to the president of the Fourth Congress the organization of an International Commission for the revision of the pharmacopœia.

The sessions were continued through six days, and all the debates and discussions appear to have been conducted in a most harmonious manner.

Over eight hundred delegates were registered. America, however, had no real representation, and England almost none. An effort was made to have the next session of the Congress held in Philadelphia; but there being no American delegates present to urge the invitation, the meeting was appointed to be held in Brussels.

The *Philadelphia Medical Times* for October 11th and 18th contains quite a full report of the proceedings of the Congress, from which we glean the above facts.

